# Biomechanical insights into Achilles tendinopathy risk and protection in runners: a large prospective study 4HAIE

**DOI:** 10.1136/bjsports-2025-110260

**Published:** 2025-12-07

**Authors:** Daniel Jandacka, Jiri Skypala, Jan Plesek, Jan Urbaczka, Milos Golian, Michal Burda, Jan Šustek, David Zahradnik, Steriani Elavsky, Vera Kristyna Jandackova, Scott Selbie, Julia Freedman Silvernail, Joseph Hamill

**Affiliations:** 1 Department of Human Movement Studies, University of Ostrava, Ostrava, Czech Republic; 2 Faculty of Physical Education and Sport, Charles University, Prague, Czech Republic; 3 Department of Diagnostic Radiology, Hospital Agel Ostrava-Vitkovice, Ostrava, Czech Republic; 4 Institute for Research and Applications of Fuzzy Modelling, University of Ostrava, Ostrava, Czech Republic; 5 Department of Epidemiology and Public Health, University of Ostrava, Ostrava, Czech Republic; 6 HAS-Motion, Kingston, Ontario, Canada; 7 Department of Kinesiology and Nutrition Sciences, UNLV, Las Vegas, Nevada, USA; 8 Dept of Kinesiology, University of Massachusetts, Amherst, Massachusetts, USA

**Keywords:** Achilles Tendon, Tendinopathy, Running

## Abstract

**Objective:**

This study aimed to evaluate whether lower limb biomechanics in runners and non-runners are risk factors for the onset of Achilles tendinopathy, and to assess the contributions of age, sex, running distance and injury history to the onset of Achilles tendinopathy.

**Methods:**

This prospective cohort study used quota sampling stratified by age, sex, region and physical activity status (runner, non-runner). Baseline assessments included questionnaires on running history and Achilles tendinopathy symptoms, running biomechanics, MRI and dual-energy X-ray absorptiometry. Participants were followed for 1 year using a Fitbit device and a custom mobile application for weekly injury reporting and orthopaedic diagnoses. Binary logistic regression identified risk factors (OR, 95% CI). Primary biomechanical variables included ankle, knee and hip kinematics and kinetics during the stance phase of running. The main outcome was a medically confirmed diagnosis of Achilles tendinopathy within 1 year.

**Results:**

Our study included 911 adults (mean age 37.7±12.5 years; 429 (47%) females; 528 (58%) runners) followed for 1 year. A higher peak ankle inversion moment (OR 0.33, 95% CI 0.17 to 0.61) during the stance phase decreased the odds of developing Achilles tendinopathy, while a lower peak ankle external rotation angle (OR 2.20, 95% CI 1.23 to 4.03) and greater running distance (OR 1.67, 95% CI 1.23 to 2.22) increased the odds of onset. The findings were consistent across the full cohort and runners.

**Conclusion:**

We identified a lower peak ankle inversion moment, a lower peak ankle external rotation angle and a greater running volume as significant predictors of the onset of Achilles tendinopathy. Targeting biomechanical factors and running volume may help prevent Achilles tendinopathy.

**WHAT IS ALREADY KNOWN ON THIS TOPIC**

**WHAT THIS STUDY ADDS**

- Our study reveals that sufficient peak external rotation levels and peak inversion moments during the stance phase of running may act as protective factors against Achilles tendinopathy development.
- This study also indicates that other biomechanical parameters, such as footfall pattern, do not significantly influence the risk of Achilles tendinopathy, challenging common recommendations to alter running footfall pattern.
- Higher weekly running volumes also significantly elevate the risk of Achilles tendinopathy.

**HOW THIS STUDY MIGHT AFFECT RESEARCH, PRACTICE OR POLICY**

- Our findings provide valuable guidance for clinicians, coaches and the running community, emphasising the need for prevention strategies that accommodate biomechanical and demographic factors to mitigate the risk of Achilles tendinopathy.
- Specifically, interventions aimed at maintaining or altering external foot rotation and ankle inversion moment during stance may offer practical, modifiable targets for Achilles tendinopathy prevention.
- These biomechanical markers could be potentially integrated into gait assessments and individualised training plans, particularly for high-mileage runners.

## Introduction

 Regular running enhances health and longevity by reducing the risk of premature mortality, cardiovascular disease and cancer.^1 2^ However, regular running can also lead to overloading of the musculoskeletal system, resulting in serious injuries. The most common running injury is chronic Achilles tendinopathy, which is characterised by pain, altered collagen structure and impaired function, thereby limiting running and sports participation and performance.^3 4^ The causes of Achilles tendinopathy in runners are considered multifactorial. One significant modifiable factor, the biomechanics of running, can be either a risk or protective factor.^5^,^7^ Due to the challenges in collecting biomechanical data, studies often have small sample sizes, making it difficult to conduct large cohort prospective studies on running biomechanics as a risk factor for Achilles tendinopathy. This also limits the ability to control for age, sex, previous injury or physical activity, which are related to Achilles tendinopathy in runners.^6^,^8^

This limited prospective evidence^8 9^ suggests certain biomechanical risk factors are associated with running. These include lower peak knee flexion and ankle dorsiflexion angles, as well as lower peak knee extensor moments in cross-country runners.^6 8^ Conversely, other findings indicate a more flexed knee angle during initial contact and at midstance of the stance phase of running, along with greater ankle dorsiflexion angle during midstance in a low-volume running (less than 51 km per week) in recreational runners and more forward-shifted touchdown in non-elite runners.^6 7^ This contradictory evidence may be due to the relatively small and specific research samples used in these prospective studies. The limitation of previous studies is that authors could not simultaneously control for prior injuries, age, sex or regular running distance (running behaviour) as factors influencing Achilles tendon structure and injury.^10 11^ Recently, a meta-analysis with strict relevance criteria found significant differences in competitive running athletes.^12^ Willwacher and associates^12^ identified increased ankle eversion angle range of motion and ankle inversion angle at initial contact as risk factors for Achilles tendinopathy, although the evidence is limited and conflicting. Another meta-analysis concluded that lower hip adduction kinematics may be a biomechanical risk factor for Achilles tendinopathy in non-elite runners, with similarly limited and conflicting evidence.^13^

Therefore, the purpose of this study was to evaluate whether lower limb running biomechanics are risk factors for the onset of Achilles tendinopathy, and to assess the contribution of age, sex, running distance and previous or current related injuries to the onset of this condition. This was investigated using the Healthy Aging in Industrial Environment Program 4 (4HAIE) large prospective cohort of runners and non-runners.^14^ We hypothesised that healthy runners with altered sagittal plane knee and ankle angles and moments during the stance phase, increased ankle eversion, strike index and hip adduction angle would be at a greater risk of developing Achilles tendinopathy during a 1-year follow-up.

## Methods

### Study design: prospective cohort study

This study presents findings from the multidisciplinary ‘Healthy Aging in Industrial Environment Program 4 (4HAIE)’ project. The study includes baseline measurements and a 1-year prospective follow-up for each participant. The prospective 4HAIE study (www.4haie.cz) has been described in detail elsewhere.^14^,^16^ In brief, one of the main goals of this study was to investigate the relationship of regular running to the incidence of running-related injuries across the life span. At baseline, all participants underwent laboratory measurements of running biomechanics and accompanying covariate measurements necessary for this study. Subsequently, participants were monitored for 1 year using Fitbit fitness trackers and a mobile phone application was used for weekly injury reporting. Participants were asked to seek orthopaedic evaluation for any suspected Achilles tendinopathy (eg, if Achilles tendon pain was reported).

### Study population and sample

The recruitment of the study participants was commissioned to a professional social science research and marketing company, selected through a publicly advertised tender. The sample was collected using quota sampling based on age, sex and physical activity status (active runner vs non-runner). Recruitment was conducted through paid advertisements on social media, field interviewers, recruitment at running events and newspaper advertisements. The inclusion criterion for regular runners was to meet the requirement of at least 150 min per week of moderate-intensity physical activity or 75 min per week of high-intensity physical activity, including running, or an equivalent combination of both, as recommended by the WHO.^17^ Participants were required to run for at least 6 weeks, with a minimum distance of 10 km per week or 6 km per week for those older than 60 years old. Non-runners had to be capable of running but did not meet the WHO recommendations for physical activity. Non-runners were included because Achilles tendinopathy is not exclusive to runners (eg, Lagas *et al*^11^ studied only runners). Our previous work showed that even inactive individuals may have altered Achilles tendon structure.^10^ Fitbit data also revealed that some non-runners had running exposure, justifying their inclusion (table 1). All participants were required to own a smartphone with internet access. For those who did not have a device, the research programme provided one. Participants were excluded from the 4HAIE study if they were people who smoked or self-reported experiencing any acute health problems in the past 6 weeks that hindered physical activity (eg, surgery, pain or injury), or any other acute illness. Other exclusion criteria were related to contraindications to MRI or dual-energy X-ray absorptiometry (DXA).

**Table 1:** Baseline characteristics of participants (N=911) without a history of Achilles tendinopathy or subjective lower limb difficulties (including both runners and non-runners) in those who developed medically diagnosed Achilles tendinopathy and those who did not during a 1-year follow-up

Variable	New Achilles tendinopathy onset group (1 year follow-up, n=23)	Remained injury-free group (n=888)	P value	Adj. p value
Age (years)	42.9±9.7	37.6±12.6	<0.05*	0.49
Sex (female/male)	7 F (30.43), 16 M (69.57)	422 F (47.52), 466 M (52.48)	0.14	1.00
Mass (kg)	73.5±14.1	74.5±14.0	0.74	1.00
Height (m)	1.75±0.09	1.74±0.08	0.67	1.00
BMI (kg/m²)	23.8±3.0	24.4±3.8	0.33	1.00
VO2max (ml/kg/min)	46.6±10.2	41.2±10.2	<0.05*	0.59
Whole body fat (%)	25.8±5.9	30.3±7.1	<0.01*	0.06
Running distance (km/week)	19 727 (9274–27 649)	5683 (0–13 849)	<0.0001*	<0.01*
Steps count (N/week)	17 116 (7801–21 741)	5355 (0–12 414)	<0.0001*	<0.01*
Speed (m/s)	2.92±0.50	2.77±0.45	0.17	1.00
Strike index (%)	10.86 (5.95–24.47)	9.32 (4.66–16.15)	0.44	1.00
Footfall pattern (RF/MF/FF)	18 RF (78.26), 4 MF (17.39), 1 FF (4)	738 RF (83.11), 102 MF (11.49), 48 FF (5)	0.56	1.00
Running frequency (steps per second)	1.66±0.28	1.59±0.25	0.21	1.00
Footwear	17 SRS (94.44), 1 M or B (5.56)	550 SRS (92.59), 44 M or B (7.41)	1.00	1.00
VIMATS MRI baseline score	95 (85–100)	100 (95–100)	0.0001*	<0.01*
VISA-A baseline score	84 (73.5–98)	94 (82–100)	<0.05*	0.75

Note: parametric variables are presented as mean±SD; non-parametric variables as median (min–max); categorical variables (eg, sex, footfall pattern) are presented as percentages. BMI refers to body mass index, a metric used to assess body composition based on an individual’s height and weight. VO_2_ max—maximum rate of oxygen consumption.^16^ SRS—standard running shoes; M or B—minimalist shoes or barefoot. Running distance (km/week)—for the injured group, this is the mean running distance from baseline until injury. For the non-injury group, this is the mean running distance from active weeks within 1 year. Steps count—average weekly sum of steps from running events. Speed—average self-selected speed determined during biomechanical testing in a laboratory environment.^15^ Strike index (SI) was estimated as initial centre of pressure location and reported as a relative foot length from the posterior calcaneus.^28^ According to SI, footfall patterns are classified as RF (rearfoot), MF (midfoot) and FF (forefoot).^28^ Footwear—specified based on the Physical Activity Survey, which included the Running Status and History questionnaire (RUNHIS).^21^ VIMATS—Vienna Morphological Achilles Tendon Score, an evaluation of Achilles tendon based on a radiologist’s MRI assessment. Individuals without Achilles tendinopathy have a VIMATS score of 95 (85–100) points, while those with Achilles tendinopathy have around 50 points.^30^ VISA-A—index of the clinical severity of Achilles tendinopathy.^22^ Individuals with Achilles tendinopathy typically have a VISA-A score of 30–70 points.^22^ Adjusted p value: the adjusted p value accounts for multiple comparisons to control the risk of obtaining significant results due to random chance.

* Statistically significant difference between groups at p < 0.05.

VISA-A, Victorian Institute of Sport Assessment-Achilles.

The 4HAIE project determined its sample size based on prior studies of running-related injuries. A systematic review by Kakouris *et al*^3^ confirmed that the most common specific overuse running injuries, such as Achilles tendinopathy, medial tibial stress syndrome, plantar fasciitis, iliotibial band syndrome and anterior knee pain, ranged from 3% to 23% in their onset. An a priori power estimation used a binary logistic regression model with three or four covariates (age, sex, running distance, plus additional biomechanical variables as a single-term covariate). A general guideline suggested having at least 10 cases with the least frequent or expected outcome for each independent variable (covariates) in the model.^18^ The lowest expected injury onset was 3% as the lowest expected specific injury onset (patellar tendinopathy and anterior knee pain). Therefore, we estimated at least 1000–1330 participants for the recruitment for the 4HAIE cohort study: (10 cases per covariate×3–4 covariates / 0.03). However, we targeted recruiting 1500 participants due to expected dropouts during the follow-up period or some missing values from baseline testing. Specifically, the onset of Achilles tendinopathy ranged from 7% to 15%, with a mean of 10.3%.^3^ For Achilles tendinopathy, the sample size estimation for a binary logistic regression, using a basic model with four covariates, would require only 570 participants. However, since 4HAIE is a multidisciplinary project, we decided to recruit more participants than the lowest necessary.

### Public and patient involvement

In the LERCO 4HAIE cohort study, we emphasised the importance of public involvement and ensured that participants’ decisions were entirely voluntary and uninfluenced, while also maintaining an ecological design to minimise any impact from the research. During the feasibility and planning stage, we conducted a pilot prospective study with runners, whose feedback on study procedures, recruitment and outcome measures directly informed the design of the main 4HAIE study. This pilot was later published^7^ and included runners who developed Achilles tendon injury, whose experience was used to refine the research questions and outcome priorities. We also consulted with regional orthopaedic specialists to ensure clinical relevance and appropriateness of the study protocol. During the screening phase, we discussed the study with individuals with a history of musculoskeletal injuries (even if not included in the main cohort), to further assess the burden and feasibility of participation. Participants spent 2 days and one night at the baseline measurement site and, over the course of a year, responded to surveys and wore Fitbit wristbands. They received regular updates on research findings and their health indicators through newsletters and attended a workshop to discuss the results. At the study’s conclusion, we organised a group physical activity event for participants and their families. Their feedback was crucial for planning the subsequent wave of measurements, which is currently underway. We have currently achieved a 67% re-enrolment rate for the second wave after 5 years. Public involvement significantly enhanced the study, providing valuable insights for both researchers and participants.

### Equity, diversity and inclusion

We selected participants from regions with contrasting environmental conditions and across socioeconomic statuses and educational levels to emphasise equity, diversity and inclusion. This included participants from a region with one of the lowest life expectancies in the Czech Republic (Moravia-Silesian region), characterised by lower socioeconomic status and prolonged high levels of air pollution.^15 16 19^ We also made efforts to foster sociodemographic inclusion and secure a balanced educational distribution across the included regions. The research team, in collaboration with the recruitment agency, implemented several targeted strategies. These strategies included offering a stipend ranging from CZK2000 to CZK2300, providing smartphones on loan for up to 1 year to participants lacking such devices, distributing small gifts and dispatching monthly newsletters to enhance engagement—particularly among individuals from lower socioeconomic backgrounds. We also made efforts to include women, including those on maternity leave, allowing children to accompany their parents in baseline measurements. This approach allowed us to address health disparities related to socioeconomic status and environmental exposure. By including diverse populations, including groups from an industrial region, we aimed to enhance the societal relevance and potential applicability of our findings across different social and environmental contexts. This commitment to equity and inclusion enhances the scientific validity of our findings and promotes health equity.

### Protocol and data collection

Each participant completed baseline socioeconomic and psychological questionnaires online via Qualtrics (Qualtrics, Provo, Utah, USA) before the laboratory visit. Baseline measurements took place over two consecutive days. On the first day, participants filled out the Physical Activity Survey, containing a physical activity questionnaire.^20^ On the second day, the Biomechanics Survey was completed, containing questions about running history, running status and injury history questionnaire^21^ and the Victorian Institute of Sport Assessment-Achilles questionnaire (VISA-A) survey as an index of the clinical severity of Achilles tendinopathy.^22^ A graded exercise test was conducted on the first day to measure the maximum oxygen consumption rate using a Blue Cherry device by Geratherm Medical AG, Germany.^16^ For comprehensive details, refer to the protocols by Elavsky *et al*^14^ and Cipryan *et al*.^16^ A standardised sleep protocol in the sleep laboratory was used.^14^

On the second day, participants underwent DXA scans for body composition and measurements for height and mass.^16^ This was followed by MRI scans to assess the structural and morphological integrity of the right Achilles tendinopathy. Each participant recruited for the study underwent a right ankle scan. The right side was selected to correspond with the limb used in biomechanical testing and to ensure consistency across participants. This approach also reduced scan time and logistical demands in this large-scale cohort. MRI data were acquired using a 1.5 T Siemens Magnetom Sempra Scanner (Siemens Healthineers, Erlangen, Germany). To obtain the highest quality images of the Achilles tendon, a 16-channel transmit/receive head coil modified for imaging the Achilles tendon was used. The ankle protocol included five sequences: SAG T1-weighted spin echo (SE), TRA T2-weighted TSE, COR T1-weighted TSE, SAG and TRA PD-weighted TSE FS and SAG T2* mapping. The imaging parameters of the sequences are described in the MRI protocol.^15^

Prior to biomechanical testing and running trials, each participant’s self-selected running speed was determined. Active runners were asked: ‘What is your usual pace when you run for 45 minutes?’ Non-runners were instructed to set a running speed that would allow them to cover the maximum possible distance if they could not estimate their pace over a 45 min period. Participants then ran overground for 2 min, during which their self-selected speed was recorded in the final 30 s using photocells.

Overground running biomechanical measurements involved eight complete overground runs on a 17 m runway, each producing a valid force plate contact with the right foot at the participant’s self-selected speed, measured by a 10-camera high-speed motion capture system (Oqus, Qualisys, Gothenburg, Sweden). Ground reaction force (GRF) data were collected using three force plates built into the 17 m long runway (Kistler, Kistler Instruments AG, Winterthur, Switzerland). Kinematics and GRF data were sampled at frequencies of 240 Hz and 2160 Hz, respectively. Overground running speed was monitored using two photocells (OPZZ, EGMedical s.r.o., Brno, Czech Republic) positioned at intervals of 3 m along the runway. A trial was deemed successful if the participant landed with their entire right foot on the force plate and maintained a speed within ±5% of their self-selected speed. For the biomechanics protocol, 32 retroreflective markers and 4 marker clusters were bilaterally attached to the pelvis, thigh, shank and foot. Additionally, markers were affixed to laboratory-neutral running shoes (Brooks Launch 5, Brooks Sport, Seattle, Washington, USA). Due to the sample size, eight experienced evaluators placed the markers, resulting in high reliability and objectivity in measuring segment lengths through repeated measurements.^23^ Although a multisegment foot model (eg, the Rizzoli model) was used during data collection, a rigid segment model was applied for analysis due to lower reliability and objectivity in the other foot subsegments. Our prior study^23^ showed that while most lower limb segments achieved excellent reliability and objectivity (intraclass correlation coefficient (ICC) >0.9), the metatarsus segment reached only moderate reliability (ICC=0.68) and the highest measurement error (standard error of measurement (SEM)=6.3%, minimal detectable change (MDC)=17.4%). For comprehensive details, refer to the protocols by Jandacka *et al*^15^ and Malus *et al*.^23^

### A 1-year follow-up and data collection on running-related injuries

During the 2-day initial measurement, participants were equipped with Fitbit Charge three or four monitors (Fitbit, San Francisco, USA) to monitor their physical activity for 1 year. Data were downloaded from the Fitbit cloud via the public API^14^ using the HealthReact platform (https://www.healthreact.eu/).

Data on running-related and physical activity injuries were collected through a combination of self-reported questionnaires and real-time injury reports. Participants were asked to report any injuries using a custom mobile application, Haieapp. Every Sunday between 16:00 and 20:00, they also completed a weekly injury survey. If there was a decrease in their usual level of physical activity, monitored by Fitbit, they received an additional injury survey.^14 24^ A physiotherapist from the 4HAIE team contacted participants by phone to gather more information about reported injuries and to recommend a clinical examination.

**Figure 1:**
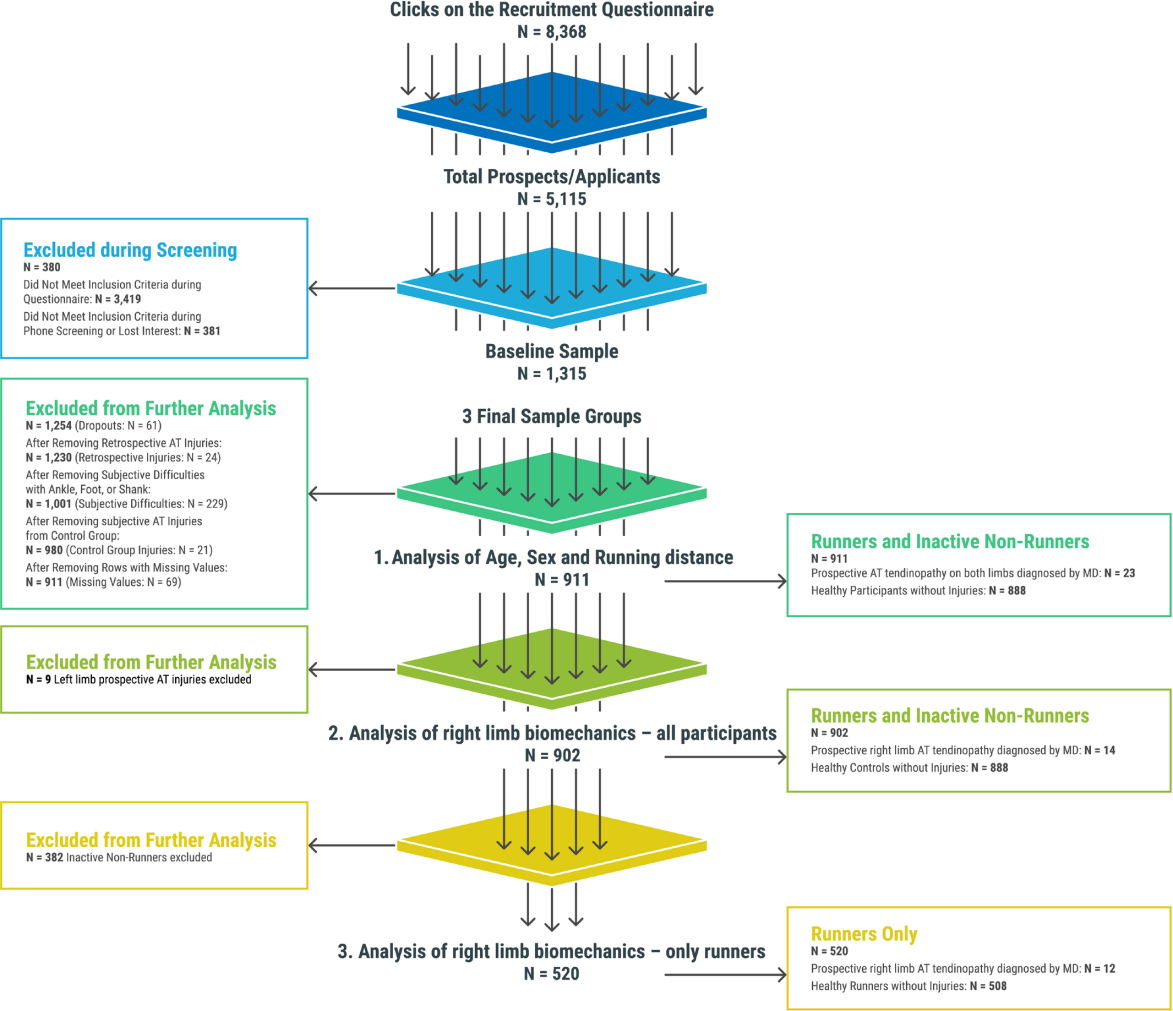
Flowchart: recruitment, screening and inclusion/exclusion process. Note: Prospective Injuries: refers to injuries that were anticipated or monitored during the 1-year study period; Healthy controls: participants who did not have any AT, foot, ankle or shank injuries during the year or retrospectively; Dropouts: participants who left the study before completion; Retrospective injuries: injuries that were identified as having occurred before the study period. Subjective difficulties: self-reported issues with specific body parts (ankle, foot or shank); Missing values: data entries that were incomplete or missing. AT, Achilles tendon; MD, medical doctor—orthopaedist.

In cases of suspected Achilles tendinopathy, participants were offered the option to consult with a study-affiliated orthopaedic specialist. However, given the geographical dispersion of the sample, they were also allowed to seek evaluation from a specialist of their own choosing. Only occurrences of Achilles tendinopathy that were formally diagnosed by a medical doctor (International Classification of Diseases, 10th revision (ICD-10) code M76.6) according to the criteria described in the Variables and Data Analysis section were included as cases in this study. Participants who did not receive a formal medical diagnosis of Achilles tendinopathy but reported symptoms consistent with Achilles tendinopathy, either retrospectively or during follow-up, including any issues involving the foot, ankle or shin, were excluded to ensure a control group free of running-related injuries (figure 1).

### Variables and data analysis

For this study, the primary dependent variable is the onset of Achilles tendinopathy within the research sample during a 1-year follow-up period. Achilles tendinopathy is the degeneration of the Achilles tendon due to overuse or repetitive stress, leading to pain, swelling and reduced function. It often results from continuous overload without proper recovery, commonly seen in regular runners.^3 4^ Therefore, Achilles tendinopathy was diagnosed by an orthopaedic specialist in accordance with ICD-10 code M76.6, based on standard clinical criteria: localised pain and/or stiffness in the Achilles tendon region, tenderness on palpation, impaired function during loading activities and imaging if necessary.

Biomechanical data were processed using Qualisys Track Manager (Qualisys, Sweden) and Visual3D software (HAS-Motion, Canada). A low-pass Butterworth filter with a cut-off frequency of 12 Hz was applied for motion data and 50 Hz for force data. Three-dimensional models of the right foot, shank, thigh and pelvis were created.^15 25^ The anterior direction of the global reference frame was aligned with the direction of running. The local segment coordinate systems were defined as the Z axis directed from the distal to the proximal end of the segment, and the Y-axis directed anteriorly. Joint angles were reported as the orientation of the distal segment relative to the proximal segment.^25^ The hip, knee and ankle joint angles were calculated using an XYZ Cardan rotation sequence (for knee and hip: X—flexion/extension about the proximal segment, Y—adduction/abduction, Z—internal/external rotation of the distal segment). For the ankle, the foot was rotated such that: X—dorsiflexion/plantar flexion, Y—internal/external rotation and Z—eversion/inversion. The foot angle was defined as the foot relative to the global reference frame using a ZYX Cardan sequence where Z is rotation about the vertical axis of the global reference frame, and X is rotation about the X axis of the foot. The three-dimensional net internal ankle, knee and hip joint moments were determined using a Newton-Euler inverse dynamics technique. Net internal ankle and knee joint moment vectors were calculated in the local coordinate system of the proximal segment.^26^ Internal joint moment refers to the tendency for the muscles and passive structures around a joint axis to create movement: for the ankle tendency, the axes represented: X axis: dorsiflexion/plantar flexion; Y axis: inversion/eversion; Z axis: internal rotation/external rotation. For the knee: X axis: extension/flexion; Y axis: adduction/abduction; Z axis: internal rotation/external rotation. The main events of running were identified based on automatic gait event identification.^27^ Mean running speed and cadence were derived from the running gait cycle for each of the eight trials. The strike index was estimated as the initial centre of pressure location and reported as a relative foot length from the posterior calcaneus.^28^ The analysed biomechanical variables of the right lower limb during the running stance phase were:

- Foot: strike index, foot angle during initial contact.
- Ankle: peak ankle dorsiflexion angle, peak ankle eversion angle, range of eversion angle, peak ankle external rotation angle, peak ankle eversion moment, peak ankle inversion moment, peak ankle plantar-flexion moment.
- Knee: peak knee flexion, knee angle during initial contact, peak knee extension moment.
- Hip: peak hip adduction angle.

For each participant, biomechanical variables were averaged across eight valid complete overground running trials to obtain a single representative value per variable.

The average weekly running distance was calculated from 1 year of physical activity data obtained via Fitbit and biomechanical lab testing. Fitbit step counts and running distance were derived by a custom script in Python with the Polars module using minute-to-minute accelerometer data. Only events with a duration of at least 8 min and a step frequency of at least 140 steps per minute in at least 3 out of every 5 consecutive minutes were included. Running distance was derived from a regression analysis model combining step frequency and speed from Fitbit GPS data with step length at the corresponding speed from biomechanical testing. Running events were identified within a 1-year follow-up for each participant. For the control group without injuries, the average weekly running distance (km/week) was derived from active weeks over the year. For those who developed Achilles tendinopathy, the average was calculated from active weeks before the injury date. An active week included at least one running event lasting at least 8 min.

The Vienna Morphological Achilles Tendon Score (VIMATS)^29 30^ was used to evaluate the baseline condition of the right Achilles tendinopathy. This assessment was conducted by an expert musculoskeletal radiologist who reviewed the MRI images. The VIMATS score evaluates Achilles tendon health based on thickness, continuity, signal intensity and associated pathologies, ranging from 0 (with 0 indicating the worst health of Achilles tendon) to 100 (indicating perfect health of Achilles tendon).

The Czech version of the VISA-A questionnaire was used. The VISA-A scores range from 0 to 100, with 100 indicating no symptoms and full physical activity. A difference of 10 points generally reflects a clinically meaningful difference.^4^ The translation to Czech was done using the back-translation method with two independent translators.^7^

### Statistical analysis

Baseline characteristics of the injury versus non-injury groups were compared using appropriate two-sample tests (independent t-tests for approximately normal data, Wilcoxon rank-sum tests for non-normal data and Fisher’s exact tests for categorical data). When multiple comparisons were made, p values were adjusted using the Holm method to control the family-wise error rate.

Before additional analysis, predictor variables were standardised (z-scored) to allow for comparability of effect sizes (ESs) across predictors. No data transformations (eg, Box-Cox, logarithmic) were applied. To identify risk factors for Achilles tendinopathy, we fitted multivariable logistic regression models. Due to imbalances in sample sizes between injured and non-injured groups, we applied Firth’s bias-reduced method^31^ and included age, sex and running distance as covariates alongside candidate biomechanical variables. First, we examined age, sex and weekly running distance as predictors of Achilles tendinopathy in the left and/or right limb within the full cohort (Analysis 1). Next, we evaluated baseline biomechanical variables as predictors of right limb Achilles tendinopathy (Analysis 2) using the subcohort with right-leg biomechanical data. Finally, we conducted a secondary analysis on the runners-only subset (Analysis 3) to verify that the identified predictors remained consistent when non-runners were excluded (see figure 1 in the results section and online supplemental appendix 1 for clarification). Single-term addition was used to assess the significance of proposed biomechanical variables in logistic regression models. All analyses were performed in R (V.4.3.1, R Core Team) using the Logistf package (V.1.26.0).

## Results

A total of 8368 attempts were made to complete the recruitment questionnaire. Of these, 5115 potential participants were identified, and 1315 were suitable for inclusion in the 4HAIE study’s measurement. Figure 1 illustrates the recruitment, screening and inclusion process for this study’s analyses.

Over the 1-year follow-up, 48 participants reported symptoms indicative of Achilles tendinopathy; however, only 30 cases were confirmed by an orthopaedic specialist as Achilles tendinopathy (the remaining 18 suspected cases were not confirmed on clinical evaluation). Of the 30 medically confirmed cases, only 23 had no prior history of left and/or right Achilles tendon injury, either subjective or objective. Among the 23 confirmed cases of Achilles tendinopathy, 10 involved the right Achilles tendon and 9 the left. The remaining cases showed involvement of both sides during the follow-up period. All individuals who had retrospective Achilles tendinopathy or who self-reported prospective injuries to the Achilles tendon, ankle, shank or foot without a formal diagnosis of Achilles tendinopathy by a medical professional were excluded from the study. Finally, 911 participants who completed the baseline laboratory measurements and 1-year follow-up of physical activity and running injuries from the 4HAIE study were eligible for the current study. The basic characteristics of the research sample are presented in table 1.

Table 1 presents the baseline characteristics of healthy participants (N=911), including runners and non-runners, categorised by the development of Achilles tendinopathy as diagnosed by a MD specialist during a 1-year follow-up period. Those who sustained injuries tended to be older (42.9±9.7 vs 37.6±12.6 years, p=0.02, adj. p=0.49), though the adjusted p value did not confirm significance (ES=0.42). They also had a lower percentage of total body fat (25.8±5.9 vs 30.3±7.1%, p<0.01, adj. p=0.06), with a moderate ES (ES=0.64). The injured group exhibited a higher median weekly sum of steps within running events (17 116 (7801–21 741) vs 5355 (0–12 414) steps, p<0.0001, adj. p<0.01), and ran longer weekly distances (19 727 (9274–27 649) vs 5683 (0–13 849) km, p<0.0001, adj. p<0.01), both with large ESs (ES=0.78 and 0.90).

Participants excluded based on the criteria in figure 1 (retrospectively examined injuries, 1-year Achilles tendinopathy related injuries and dropout cases) did not differ from those included in this study with respect to age (39.1±12.6 vs 37.7±12.5 years, adj. p values ≥0.05), sex (F: 44.6% vs 47.1%, M: 55.5% vs 52.9%, adj. p values ≥0.05) or weekly running distance (11 982±15 057 vs 9974±12 636 km, adj. p values ≥0.05).

At the beginning of the study, all participants were free of subjective Achilles tendinopathy symptoms, as confirmed by the comparison VISA-A baseline scores. The injured group had a median VISA-A score of 84 (73.5–98) points, compared with 94 (82–100) in the non-injured group (adj. p=0.76). Although this difference is not statistically significant, it may reflect a possible subclinical variation rather than manifest tendinopathy, as symptomatic individuals typically have scores between 30 and 70.^22^ The Achilles tendinopathy group had a median VIMATS score of 95 (85–100), while the non-injured group had 100 (95–100) (adj. p<0.01).

### Prospective association between age, sex, running distance at baseline and the onset of Achilles tendinopathy during 1-year follow-up (911 runners and non-runners, 23 new Achilles tendinopathy cases observed over a 1-year follow-up, occurrence noted on either the left or right Achilles tendon)

The logistic regression model assessed whether age, sex and running distance predicted odds of Achilles tendinopathy. The results are summarised in table 2.

**Table 2:** Multivariable logistic regression model including age, sex and running distance entered simultaneously

Variable	Estimate	Error	OR	95% CI for OR	P value
Age (years)	0.38	0.23	1.46	0.93 to 2.32	0.10
Sex (reference: female)	0.49	0.45	1.62	0.68 to 4.20	0.28
Running distance derived from Fitbit during 1-year follow-up (km/week)	0.51	0.15	1.67	1.23 to 2.22	<0.01*

* Statistically significant association (p < 0.05).

**Figure 2:**
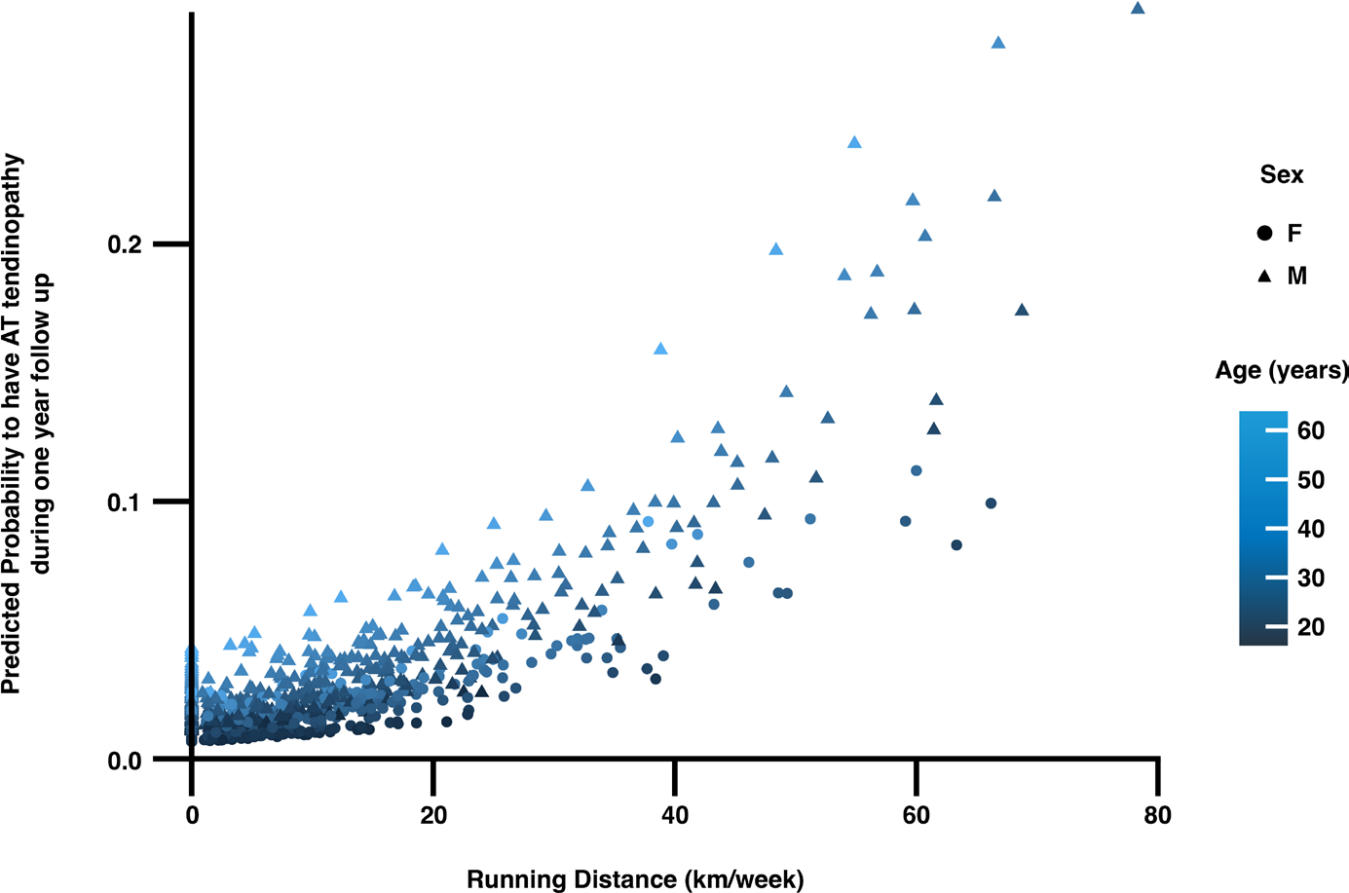
Logistic regression model. Higher weekly running distances during 1-year follow-up increase the likelihood of developing Achilles tendinopathy when controlling for sex and age. AT, Achilles tendon.

Higher running distance was associated with increased odds of Achilles tendinopathy. The logistic regression model showed that with each increase in weekly running distance by one standard deviation (ie, 12.5 km/week), the odds of Achilles tendinopathy increased by 67% (OR 1.67, 95% CI 1.23 to 2.22, p<0.01) (figure 2.)

The VISA-A score was a significant predictor of Achilles tendinopathy. In the model including age, sex and running distance, each 1 SD decrease in VISA-A score (14 points) was associated with an 85% increase in the odds of Achilles tendinopathy (OR for 1 SD increase=0.54, so for 1 SD decrease=1/0.54 ≈ 1.85, 95% CI 0.36 to 0.82, p=0.005). Similarly, each 1 SD decrease in VIMATS total score (6 points) was associated with a 43% increase in the odds of Achilles tendinopathy (OR for 1 SD increase=0.70, so for 1 SD decrease=1/0.70 ≈ 1.43, 95% CI 0.54 to 0.93, p=0.02). In the full model including both scores and covariates (table 2), higher running distances remained a significant predictor, with ORs ranging from 1.67 to 1.96 and p values from 0.001 to <0.001 across models including VIMATS and VISA-A scores.

During the 1-year follow-up, the onset of new Achilles tendinopathy was observed in 3.8% of runners (20 out of 528) and in only 0.8% of inactive non-runners (3 out of 383). Fisher’s exact test showed this difference was statistically significant (p<0.01).

### Baseline biomechanical parameters in both runners and non-runners and association with odds of subsequent onset of Achilles tendinopathy (902 participants, 14 new right Achilles tendinopathy cases observed over a 1-year follow-up)

Adding biomechanical variables from earlier studies to the basic model showed that peak ankle external rotation angle and peak ankle inversion moment during stance were significant predictors of Achilles tendinopathy, with notable differences in direct comparisons (see onlinesupplemental appendices 2^ 3^).

The logistic regression analysis, controlling for sex, age and running distance, revealed that for each increase by one SD in the inversion moment (SD=17 Nm), the odds of Achilles tendinopathy injury decreased by 67% (OR 0.33, 95% CI 0.17 to 0.61, p<0.001). In the extended model including VISA-A score, this association remained significant (OR 0.30, 95% CI 0.15 to 0.57, p<0.001). Similarly, for each decrease by one SD in external rotation angle (SD=6° a shift toward less negative values, representing less external rotation), the odds of Achilles tendinopathy increased by 120% (OR 2.20, 95% CI 1.23 to 4.03, p<0.01). This association also remained significant in the model adjusted for VISA-A (OR 2.35, 95% CI 1.29 to 4.35, p<0.01).

To ensure thoroughness, online supplemental appendix 3 presents a direct comparison of baseline biomechanical parameters of the right lower limb during the stance phase between participants who developed right Achilles tendinopathy within 1 year and those who remained uninjured. The peak ankle inversion moment during the stance phase was significantly lower in the Achilles tendinopathy group by 14.39 Nm (pooled SD=16.97, adjusted p value <0.01, ES=0.85). The peak ankle external rotation angle was significantly lower in the Achilles tendinopathy group by 4.10 degrees (pooled SD=5.52, adjusted p value <0.05, ES=0.74).

### Baseline biomechanical parameters in runners only and odds of subsequent onset of Achilles tendinopathy (520 runners, 12 new right Achilles tendinopathy cases observed over a 1-year follow-up)

**Figure 3:**
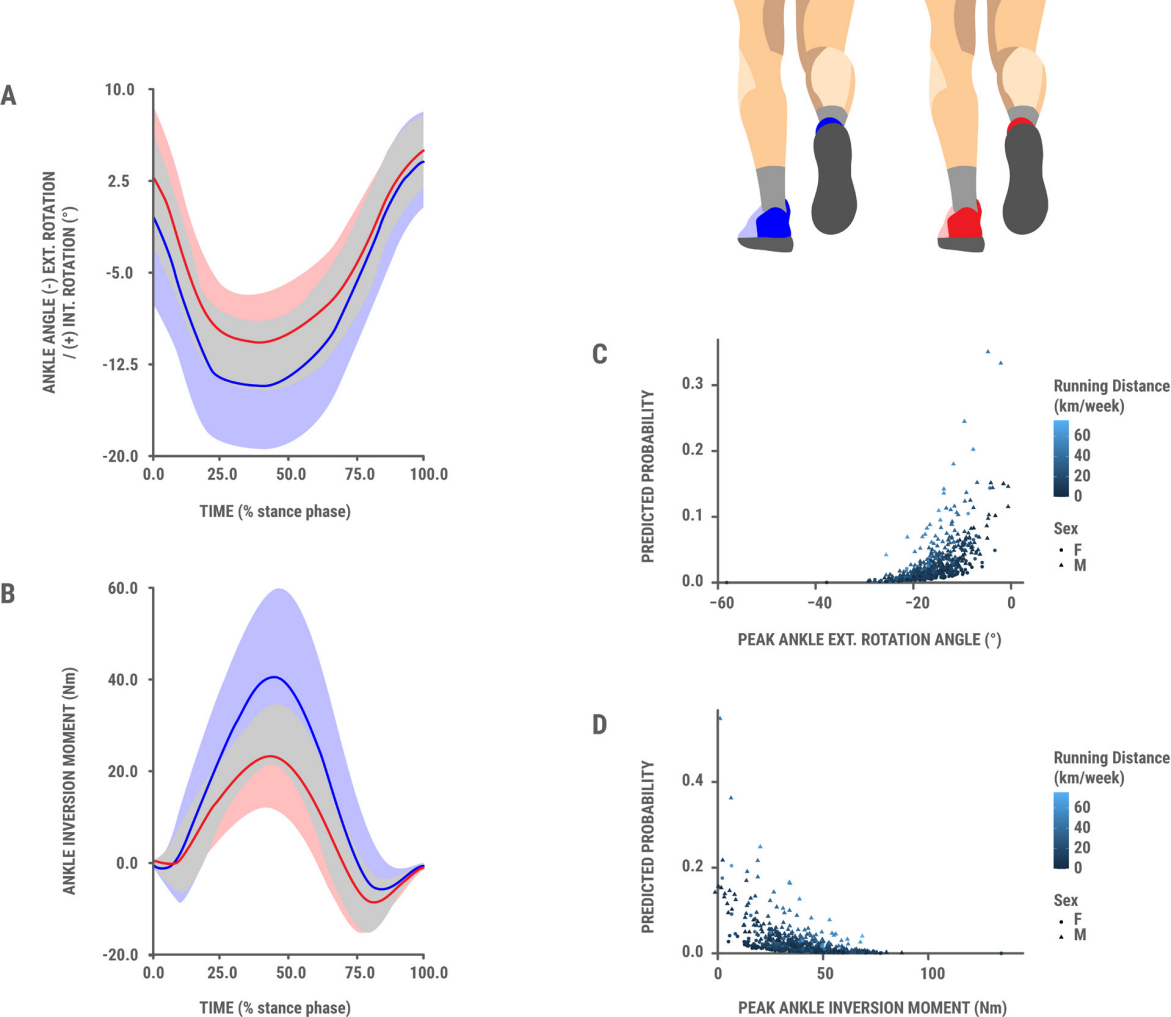
Baseline comparison of peak ankle. (**A**) Int/external rotation angles and (**B**) inversion/eversion moments during stance phase among initially uninjured runners who subsequently developed AT (n=12), shown in red (bold mean and shaded SD), and injury-free controls (n=12), shown in blue. To make pairs, injury-free controls were selected retrospectively after 1-year follow-up from the uninjured group based on age, sex, weekly running distance, foot strike index, height and weight. (**C and D**) Baseline peak stance phase ankle (**C**) external rotation angle and (**D**) inversion moment and the odds of developing AT over 1 year (n=520). The figures (**C and D**) show that greater peak stance phase ankle (**C**) external rotation angle and (**D**) inversion moment are both associated with lower odds of AT onset. Predicted probabilities are from a logistic regression model adjusted for sex, age and running distance. AT, Achilles tendinopathy

In the logistic regression analysis conducted on runners only, like the analysis in runners and non-runners (Analysis 2), lower peak ankle external rotation angles significantly predicted higher odds of Achilles tendinopathy, while higher peak ankle inversion moments were significant predictors in reducing the odds of Achilles tendinopathy (figure 3). With each decrease in external ankle rotation angle by one standard deviation (ie, 6° shift to less negative values, representing less external rotation), the odds of Achilles tendinopathy increased by 115% (OR 2.15, 95% CI 1.12 to 4.14, p<0.05). In addition, with each increase in ankle inversion moment by one standard deviation (ie, 17 Nm), the odds of Achilles tendinopathy decreased by 64% (OR 0.36, 95% CI 0.18 to 0.68, p<0.01).

## Discussion

The aim of this study was to identify biomechanical risk factors for the onset of Achilles tendinopathy in runners and non-runners. Specifically, we evaluated whether lower limb biomechanics were prospectively associated with the odds of developing medically diagnosed Achilles tendinopathy within 1 year of testing while controlling for age, sex, running distance and injury history. Based on previous predominantly retrospective studies, as well as some prospective studies with limited sample sizes, we hypothesised that runners with altered knee and ankle angles and moments, increased ankle eversion, strike index and hip adduction angle are more likely to develop Achilles tendinopathy within a year. Our findings indicated that reduced peak ankle external rotation angles and lower peak inversion moments during the stance phase of running increase the likelihood of Achilles tendinopathy.

A 6° reduction in peak external foot rotation angle was linked to approximately a 120% increase in the odds of Achilles tendinopathy, suggesting that less external foot rotation during stance might negatively impact how the Achilles tendon distributes load. Conversely, a 17 Nm increase in peak ankle inversion moment correlates with a 67% decrease in the odds of Achilles tendinopathy, highlighting the importance of ankle control and inversion strength in reducing stress on the tendon. These findings suggest that lower ankle inversion moments and lower peak external rotation angles are independently associated with increased odds of Achilles tendinopathy. Given the prospective nature of this study, these biomechanical differences may reflect causal mechanisms.

### Biomechanical Achilles tendinopathy risk and protection

Recent meta-analyses have identified increased eversion during the stance phase as a biomechanical risk factor for Achilles tendinopathy in runners.^12^ An analysis encompassing 13 primarily retrospective studies demonstrated inconsistent evidence across varied runner profiles underscoring the necessity for further research. Contrary to traditional views positing increased eversion as a risk factor for Achilles tendinopathy,^32 33^ our study suggests that adequate eversion, along with greater maximal inversion moments, might confer a protective effect against Achilles tendinopathy by facilitating the absorption of mechanical energy during the stance phase. One plausible explanation is that the optimal range of eversion and greater inversion moments mitigate impact forces during running in both the frontal and sagittal planes. Muscles such as the soleus and gastrocnemius, which primarily function as ankle plantar flexors and knee flexors, significantly load the Achilles tendon predominantly in the sagittal plane.^34^ Force absorption through natural ankle eversion and inversion moments in the frontal plane during running aids in the dissipation of energy, thereby preventing the transfer of the entire load to the sagittal plane, where the Achilles tendon experiences the greatest strain. However, we can also hypothesise that reduced peak inversion moments can compromise the stability of the foot and ankle during running, leading to compensatory movements that may increase strain on the Achilles tendon. Although our study did not find increased eversion among injured runners, lower inversion moments correlated with increased eversion, which may support previous evidence suggesting that increased eversion during the stance phase may be associated with risk for Achilles tendinopathy in specified groups of runners.^12^

Moreover, reduced peak ankle external rotation angles may increase the odds of Achilles tendinopathy due to altered torsional stress on the Achilles tendon, which could result in microtrauma and inflammation, or inappropriate/insufficient eccentric loading, contributing to the development of Achilles tendinopathy.^35^ These findings raise the hypothesis that increased external foot rotation may exert a protective effect by altering the vector of force transmission within the Achilles tendon, thereby potentially reducing mechanical load on the gastrocnemius lateralis subtendon. Given that stiffness of the gastrocnemius lateralis is selectively reduced in individuals with Achilles tendinopathy,^36^ such redistribution of load may mitigate stress in this region. Although direct evidence linking foot rotation to subtendon-specific loading during running is currently lacking, this mechanism may help explain prospective observations associating greater external foot rotation with a reduced risk of Achilles tendinopathy in runners. In addition to biomechanical considerations, neuromuscular control may also play a role in modulating tendon stress. Notably, recent neurophysiological data show that external foot rotation reduces neural drive to the gastrocnemius lateralis during submaximal isometric plantarflexion in a seated position,^37^ which may contribute to lower mechanical demand on its subtendon. This finding implies that foot rotation may influence not only mechanical loading patterns but also neuromuscular control strategies. Future studies should investigate the subtendon-level biomechanical and neuromuscular consequences of foot orientation during running.

In our study, we further identified that other biomechanical parameters, previously recognised in retrospective studies or prospective studies with limited sample sizes, do not significantly influence the likelihood of developing Achilles tendinopathy.^6^,^812 13^ These parameters include running speed, strike index, cadence, peak dorsiflexion angle, foot angle at initial contact, peak plantar-flexion moment, peak hip adduction angle, knee flexion angle at initial contact, peak knee flexion angle during stance and peak knee extension moment. Specifically, recommendations to change the type of footfall pattern from forefoot to heel strike or to aim for a midfoot strike to reduce the risk of chronic Achilles tendinopathy during running are common in mass media as well as studies in professional literature with retrospective or cross-sectional designs. For example, one retrospective study on runners with previous injuries identified that running with a midfoot strike increases the chance of developing Achilles tendinopathy by 2.3 times.^5^ However, the current prospective study did not find evidence to support this claim, which may stem from injury-related adaptations. We showed that the distribution of footfall patterns was similar in both the Achilles tendinopathy and non-injured groups. The results of our study thus demonstrated that the type of footfall pattern (rearfoot, midfoot or forefoot) is not associated with a higher risk of developing Achilles tendinopathy.

Our study’s rigorous selection criteria, which excluded runners with prior Achilles tendon injuries, may have enabled us to discern the mechanical causes of Achilles tendinopathy rather than its consequences.^38^ However, previous findings that link midfoot footfall pattern or greater eversion during the stance phase with Achilles tendinopathy in retrospective studies may reflect an adaptive mechanism aimed at protecting an already compromised Achilles tendinopathy rather than a causative factor.^5^

### Age, sex, running distance and Achilles tendinopathy risk and protection

Our research further underscores that Achilles tendinopathy is a prevalent injury among runners. The onset of this condition increased in individuals with higher weekly running volumes prior to the injury. In contrast, participants who were not prospectively injured consistently maintained lower running volumes throughout the entire year of observation and/or were usually non-runners. An increase of 12.5 km in weekly running distance was associated with a 64% increase in the odds of Achilles tendinopathy, indicating the importance of monitoring training volume, especially for high-mileage runners. Previous research has indicated that running distances exceeding 40 km per week in middle-aged runners lead to disruptions in collagen and water content in the Achilles tendon, as indirectly measured by MRI.^10^ The study found that running less than 40 km per week was linked to healthier tendon structures compared with higher running volumes or inactivity, potentially reducing the risk of future injuries. The concept of collagen supercompensation indicates that the Achilles tendon requires approximately 1.5–3 days for collagen regeneration following physical activity.^39^ Extensive weekly running volumes may prevent adequate time for the supercompensation phase, thus increasing the likelihood of developing Achilles tendinopathy.

Although age was not a statistically significant predictor in our adjusted models, runners in the injured group tended to be older (ES=0.41). Therefore, runners, particularly those of advanced age, should exercise with caution when increasing their running volume whether for competition or fitness purposes. The structure and mechanical properties of the Achilles tendon change with age, impacting its function and ability to adapt to physical stress.^40 41^ However, this relationship may be non-linear, suggesting that there could be an optimal volume or load for each individual at a given age that promotes a healthy Achilles tendon structure.^10^ Caution when increasing running volume is essential for both sexes. According to our study, sex is not a statistically significant factor for the risk of developing Achilles tendinopathy. Although men exhibit a higher OR, the CI indicates that the difference between sexes is not significant. This finding contrasts with some previous research suggesting that males may be at higher risk.^5^ However, it highlights the need for further research to explore the potential sex differences in tendon pathology. Therefore, it is crucial for clinicians and trainers to consider these factors when designing training programmes and preventive strategies for runners, particularly those in advanced age or those with high running volume.

### Strengths and limitations

One of the primary strengths of this study lies in its prospective study design and its extensive scope, incorporating both running and non-running populations, with a robust consideration for biomechanics while objectively controlling for physical activity, sex, age and previous and subsequent injuries. This allowed us to assess Achilles tendinopathy risk factors beyond running populations and objectively capture running exposure even among non-runners, addressing a key limitation of previous studies.^11^ Unlike prior prospective studies that relied on subjective injury identification,^7^ this study used physician diagnoses for the Achilles tendinopathy injury. Furthermore, Achilles tendon health was confirmed through both questionnaires and MRI scans at baseline, interpreted by a radiologist. Another notable strength is the year-long monitoring facilitated by innovative IT technologies, which enabled the minimising of recall bias in reporting Achilles tendinopathy injuries as well as tracking subsequent and historical injuries. The multidisciplinary approach not only allowed for a comprehensive biomechanical analysis of both active runners and non-runners but also integrated physiological factors into the research. This rigorous methodology, combined with the advanced diagnostic tools and technology, underscores the robustness and reliability of the study’s findings.

One limitation of this study is the absence of a multisegment foot model,^26^ which could have provided a more detailed analysis of biomechanical factors related to eversion. However, our foot model allows us to describe the relationships between kinematics and kinetics and understand the connections between ankle eversion and inversion moments. This limitation may affect the generalisation of our findings when compared with other studies that have used different biomechanical foot models pertaining to the protective and risk factors for the development of Achilles tendinopathy and other injuries.^26^ Participants were tested in standardised laboratory shoes, presenting both strength and limitations. The uniform footwear provided consistent testing conditions, helping to understand ageing effects after 5 years. The second wave of data collection is currently underway after 5 years (2024–2026), which can provide unique insights into the long-term effects of ageing on individuals. However, this factor may represent a limitation since participants did not wear our neutrally cushioned running shoes during the 1-year follow-up, potentially affecting the ecological validity of the results.

This study encounters the limitation of analysing discrete biomechanical variables (ie, peak eversion angle rather than eversion as a continuous variable during the stance phase), which leads to the loss of some information from the entire stance phase signal during running. However, our approach enables the use of statistical models such as logistic regression, which are not well-suited for continuous time-series data. While time-resolved methods such as Statistical Parametric Mapping (SPM) offer richer temporal insights^42^ and are typically applied to continuous outcomes and not to binary endpoints such as injury occurrence. Therefore, we used waveform data only illustratively (figure 3). Future studies may consider combining SPM with continuous outcomes or hybrid models to explore waveform-level predictors of injury risk. This interdisciplinary collaboration could lead to more comprehensive strategies for injury prevention, and such integration could pave the way for applications using artificial intelligence technologies and automatic injury or overload risk prediction.

Another limitation involves the geographical and demographic restriction of participant selection to ages 18–65, which may impact the generalisability of the findings to a broader population. Our study focused on runners and non-runners within a specific geographical area, which implies that the results may not be fully applicable to other demographic groups or regions with different training habits and living conditions. Moreover, studies on healthy living often involve motivated, healthier individuals, which skews selection. Additionally, while Achilles tendinopathy diagnoses were conducted by specialists and confirmed via MRI at baseline, there remains the possibility that some cases may have been overlooked or misdiagnosed, potentially impacting the overall results. Another source of potential bias may arise from subjective responses to questionnaires regarding running habits and injury history. Other limitations include the potential for residual confounding from unmeasured factors that may be relevant to Achilles tendinopathy or independent variables. These limitations are common to all observational studies.

## Conclusion

Our study presents evidence that sufficient peak external rotation of the foot and peak ankle inversion moments during the stance phase of running may serve as a protective factor against Achilles tendinopathy. This finding challenges the conventional approach of limiting non-rearfoot-fall patterns in runners. Moreover, our research indicates that higher weekly running distances significantly elevate the risk of Achilles tendinopathy. Additionally, Achilles tendinopathy emerged as a typical condition among runners, as the control group exhibited significantly lower onset rates compared with runners. While age did not emerge as a statistically significant predictor, affected runners tended to be older. Targeting modifiable biomechanical factors and running volume may help prevent Achilles tendinopathy, particularly in runner profiles more prevalent among those affected. These findings support the integration of biomechanical screening and individualised running distance management into injury prevention strategies, particularly for high-volume runners.

## Supplementary material

10.1136/bjsports-2025-110260 online supplemental file 1

10.1136/bjsports-2025-110260 online supplemental file 2

10.1136/bjsports-2025-110260 online supplemental file 3

## Data Availability

Data are available upon reasonable request.
