# Supplementary material for: Biomechanical insights into Achilles tendinopathy risk and protection in runners: a large prospective study 4HAIE
Source: Br J Sports Med. 2025 Dec 7;60(3):e110260. doi: 10.1136/bjsports-2025-110260 (PMC13018850; doi:10.1136/bjsports-2025-110260)
Supplement: online supplemental file 1 [file bjsports-60-3-s001.docx]

***Prospective association between age, sex, and baseline running distance and the onset of Achilles tendinopathy at follow-up* (Analysis 1):**

Initially, using binary logistic regression we examined whether age, sex, and running distance could predict the likelihood of Achilles tendinopathy occurrence in 911 participants (comprising both runners and inactive non-runners), among whom 23 new cases were diagnosed by a MD specialist over a one-year follow-up period. The occurrence was noted on either the left or right Achilles tendon (See Figure 1 Flowchart). Moreover, in the logistic regression analysis, we progressively controlled for subjective Achilles tendinopathy complaints using the VISA-A score and the morphology of the Achilles tendon, which was assessed by a radiologist using MRI and VIMATS.

***Baseline biomechanical parameters in runners and non-runners and their association with the likelihood of subsequent onset of Achilles tendinopathy.* (Analysis 2):**

In the subsequent analysis we focused on comparing biomechanical variables and related factors between individuals who sustained Achilles tendinopathy over one year and the uninjured population. However, not all Achilles tendinopathy cases could be utilized due to economic and logistical-technical reasons, as MRI measurements and running biomechanics evaluations were conducted only on the right lower limb. Thus, this analysis included 902 runners and inactive individuals. We used T-test to compare the injured group of 14 participants, diagnosed by an MD specialist over a one-year follow-up period, and non-injured control group of 888 participants. We used a single-term addition approach to assess the significance of biomechanical variables in a logistic model of Achilles tendinopathy. Holm's method adjusted p-values for multiple comparisons. Firth's bias-reduced logistic regression evaluated if right limb biomechanics predicted Achilles tendinopathy within a year, considering age, sex, running distance, and injury history.

***Baseline biomechanical parameters in runners and their association with the likelihood of subsequent onset of Achilles tendinopathy* (Analysis 3):**

Logistic regression was conducted to examine whether running biomechanics could predict the likelihood of Achilles tendinopathy occurrence in 520 runners among whom 12 new cases were diagnosed by a MD specialist over a one-year follow-up period.

To visualize baseline differences in continuous biomechanical variables during the stance phase of running (Figure 3), we matched 12 runners who developed Achilles tendinopathy at follow-up with 12 injury-free controls. Matching was based on age, sex, weekly running distance, foot strike index, height, and mass, with the closest possible alignment across these variables. Additionally, we visualized the predicted probability of Achilles tendinopathy occurrence using logistic regression.
