# Supplementary material for: Biomechanical insights into Achilles tendinopathy risk and protection in runners: a large prospective study 4HAIE
Source: Br J Sports Med. 2025 Dec 7;60(3):e110260. doi: 10.1136/bjsports-2025-110260 (PMC13018850; doi:10.1136/bjsports-2025-110260)
Supplement: online supplemental file 2 [file bjsports-60-3-s002.docx]

**Appendix 2 -Table 3.** Effect of adding individual biomechanical parameters to a base model (age, sex, and running distance) predicting Achilles tendinopathy onset over 1-year follow-up (902 participants, 14 new Achilles tendinopathy cases for one year follow-up). Wald statistics and adjusted p-values are reported.

| **Variable** | **Estimate** | **Error** | **Wald** | **p-value** | **Adj. p-value** |
| --- | --- | --- | --- | --- | --- |
| Running speed | -0.02 | 0.29 | 0.0 | 0.96 | 1.00 |
| Strike index | 0.21 | 0.22 | 0.7 | 0.40 | 1.00 |
| Strides per second | 0.21 | 0.3 | 0.4 | 0.52 | 1.00 |
| Foot angle at initial contact | -0.12 | 0.24 | 0.2 | 0.66 | 1.00 |
| Peak ankle dorsiflexion angle | 0.05 | 0.26 | 0.03 | 0.86 | 1.00 |
| Peak ankle eversion angle | 0.31 | 0.21 | 1.37 | 0.24 | 1.00 |
| Range of eversion angle | -0.12 | 0.24 | 0.21 | 0.64 | 1.00 |
| Peak ankle external rotation angle | 0.79 | 0.28 | 6.99 | <0.01* | 0.15 |
| Peak ankle inversion moment | -1.11 | 0.31 | 13.24 | <0.001* | <0.01* |
| Peak ankle plantarflexion moment | 0.22 | 0.33 | 0.38 | 0.54 | 1.00 |
| Knee angle at initial contact | 0.23 | 0.25 | 0.76 | 0.38 | 1.00 |
| Peak knee stance flexion angle | 0.3 | 0.27 | 1.08 | 0.30 | 1.00 |
| Peak knee extension moment | -0.9 | 0.37 | 5.79 | 0.02* | 0.26 |
| Peak hip adduction angle | 0.16 | 0.28 | 0.28 | 0.60 | 1.00 |

*Indicates statistical significance
