# Supplementary material for: Biomechanical insights into Achilles tendinopathy risk and protection in runners: a large prospective study 4HAIE
Source: Br J Sports Med. 2025 Dec 7;60(3):e110260. doi: 10.1136/bjsports-2025-110260 (PMC13018850; doi:10.1136/bjsports-2025-110260)
Supplement: online supplemental file 3 [file bjsports-60-3-s003.docx]

**Table 4** Comparative analysis of baseline biomechanical parameters of right lower limb during the stance phase of participants who sustained a right Achilles tendon during the one-year follow-up period and who were non-injured.

| Variable | New Achilles Tendinopathy Onset Group (one-year follow-up, N=14) | Remained Injury-Free Group (N=888) | p-value | adj. p-value |
| --- | --- | --- | --- | --- |
| Running speed (m/s) | 2.93 ± 0.49 | 2.77 ± 0.45 | 0.25 | 1.00 |
| Running frequency (steps per second) | 1.70 ± 0.27 | 1.59 ± 0.25 | 0.15 | 1.00 |
| Footwear: SRS (%) / MorB (%) | SRS: 90.0 / M or B: 10.0 | SRS: 92.6 / M or B: 7.4 | 0.54 | 1.00 |
| **Foot** | | | | |
| Strike index (%) | 10.10 [6.57–34.00] | 9.32 [4.66–16.15] | 0.39 | 1.00 |
| Foot angle at initial contact sagittal (°) | 77.9 ± 10.4 | 79.4 ± 9.8 | 0.62 | 1.00 |
| **Ankle** | | | | |
| Peak ankle dorsiflexion angle (°) | 85.6 ± 3.8 | 86.0 ± 4.2 | 0.68 | 1.00 |
| Peak ankle eversion angle (°) | -7.0 ± 4.3 | -7.8 ± 4.3 | 0.48 | 1.00 |
| Range of eversion angle (°) | 10.9 ± 3.9 | 10.4 ± 3.6 | 0.68 | 1.00 |
| Peak ankle external rotation angle (°) | -11.5 ± 3.7 | -15.6 ± 5.5 | <0.01* | 0.04* |
| Peak ankle plantar-flexion moment (Nm) | -172.0 ± 51.2 | -167.1 ± 40.8 | 0.72 | 1.00 |
| Peak ankle eversion moment (Nm) | -10.2 ± 6.4 | -5.8 ± 5.7 | 0.02* | 0.67 |
| Peak ankle inversion moment (Nm) | 23.1 ± 10.6 | 37.5 ± 17.1 | <0.001* | <0.01* |
| **Knee** | | | | |
| Knee angle at initial contact (°) | -10.2 ± 6.2 | -11.4 ± 5.1 | 0.46 | 1.00 |
| Peak knee stance flexion angle (°) | -38.7 ± 6.4 | -40.9 ± 5.3 | 0.22 | 1.00 |
| Peak knee extension moment (Nm) | 171.4 ± 57.4 | 190.0 ± 50.4 | 0.25 | 1.00 |
| **Hip** | | | | |
| Peak hip adduction angle (°) | 12.6 ± 3.3 | 12.7 ± 4.1 | 0.92 | 1.00 |

***Note:*** *Parametric variables are presented as mean ± standard deviation (SD); non-parametric variables as median [min–max]; categorical variables (e.g. footfall pattern) are presented as percentages.* Abbreviations: SRS: standard running shoes, M – minimalist shoes, B – barefoot. Significant differences were found in two biomechanical variables between the AT group and the non-injury group. We tested only variables previously identified as potential risk factors in earlier studies. The peak ankle inversion moment during stance phase was significantly lower in the AT group by 14.39 Nm (Pooled SD = 16.97, adjusted p-value = 0.007, ES = 0.85). The peak ankle external rotation angle was significantly lower in the AT group by 4.10 degrees (Pooled SD = 5.52, adjusted p-value = 0.04, ES = 0.74).
